# Applying conventional and cell-type-specific CRISPR/Cas9 genome editing in legume plants

**DOI:** 10.1007/s42994-024-00190-4

**Published:** 2024-12-16

**Authors:** Jin-Peng Gao, Yangyang Su, Suyu Jiang, Wenjie Liang, Zhijun Lou, Florian Frugier, Ping Xu, Jeremy D. Murray

**Affiliations:** 1 CAS-JIC Centre of Excellence for Plant and Microbial Science (CEPAMS), CAS Center for Excellence in Molecular and Plant Sciences, Chinese Academy of Sciences, Shanghai, 200032 China; 2 Crop Science Centre, Department of Plant Sciences, University of Cambridge, Cambridge, CB3 0LE UK; 3 Institute of Plant Sciences of Paris-Saclay (IPS2), CNRS, INRAE, Université Paris-Saclay, Gif Sur Yvette, 91190 France; 4 Shanghai Engineering Research Center of Plant Germplasm Resource, College of Life Sciences, Shanghai Normal University, Shanghai, 200234 China; 5 University of Chinese Academy of Sciences, Beijing, 100049 China; 6 Cell and Developmental Biology, John Innes Centre, Norwich, NR4 7UH UK

**Keywords:** CRISPR/Cas9, Genome editing, Nodulation, Symbiosis, Genetic transformation, Tissue-specific promoter

## Abstract

The advent of genome editing technologies, particularly CRISPR/Cas9, has significantly advanced the generation of legume mutants for reverse genetic studies and understanding the mechanics of the rhizobial symbiosis. The legume–rhizobia symbiosis is crucial for sustainable agriculture, enhancing nitrogen fixation and improving soil fertility. Numerous genes with a symbiosis-specific expression have been identified, sometimes exclusively expressed in cells forming infection threads or in nitrogen-fixing nodule cells. Typically, mutations in these genes do not affect plant growth. However, in some instances, germline homozygous mutations can be lethal or result in complex pleiotropic phenotypes that are challenging to interpret. To address this issue, a rhizobia-inducible and cell-type-specific CRISPR/Cas9 strategy was developed to knock-out genes in specific legume transgenic root tissues. In this review, we discuss recent advancements in legume genome editing, highlighting the cell-type-specific CRISPR system and its crucial applications in symbiotic nitrogen fixation and beyond.

## Introduction

Plants directly and indirectly provide food, energy sources and other consumable resources for human life (Martin et al. 2011). Among these, legumes play a crucial role in both ecological and agricultural systems, significantly contributing to the biological nitrogen fixation through their symbiotic association with nitrogen-fixing soil bacteria known as rhizobia (Oldroyd and Leyser 2020; Xu and Wang 2023). This symbiotic relationship not only enhances soil fertility but also reduces the need for chemical fertilizers, promoting more sustainable agricultural practices (Jhu and Oldroyd 2023). Understanding beneficial microbial associations and improving leguminous crops is, therefore, both critical and urgent.

The establishment of symbiosis between legumes and rhizobia involves a complex molecular dialogue and the activation of symbiosis signaling pathways in plants, resulting in two coordinated developmental processes: rhizobial infection and nodule organogenesis (Roy et al. 2020). In most legumes, rhizobia enter roots through root hairs via plant-made structures known as infection threads, which are tubular invaginations of the host cell wall and plasma membrane that grow across cells and are colonized by rhizobia (Gao et al. 2024; Liang et al. 2021). The progressing infection threads ramify into the underlying nodule primordium, after which the rhizobia are released from infection threads into nodule cells where they fix atmospheric nitrogen into ammonia (de Carvalho-Niebel et al. 2024; Su et al. 2023).

Over the past decades, significant progress has been made in understanding molecular mechanisms underlying the nodule symbiosis and other critical aspects of legume biology (Roy et al. 2020; Wang et al. 2022). This progress has been largely driven by advancements in genetic and genomic technologies, which have enabled detailed studies of gene function and regulation through the generation of mutant alleles (Espina et al. 2018; Penmetsa and Cook 2000; Perry et al. 2003; Szczyglowski et al. 1998). Effective mutagenesis approaches, such as ethyl methane sulfonate (EMS), ionizing radiation, and retrotransposon element insertions, have been instrumental in identifying symbiotic genes. Notable resources, such as the *Lotus japonicus Retrotransposon 1* (*LORE1*) insertion mutants (Fukai et al. 2012; Urbanski et al. 2012) and the *Medicago truncatula Tnt1* retrotransposon insertion population (Sun et al. 2019; Tadege et al. 2008), facilitate both forward and reverse genetic studies.

Despite these successes, mutagenesis approaches face challenges, particularly the complication of identifying causal mutations due to multiple insertions within individual lines and functional redundancies between closely related genes. This complexity can hinder the precise determination of genotype–phenotype relationships, necessitating advanced techniques to isolate and study-specific genetic alterations. RNA interference (RNAi) is widely used in symbiosis research to circumvent functional redundancy (Qiao et al. 2023). However, the RNAi approach suffers from high variability in knockdown efficiencies across genes and plant species. In addition, RNAi can lose effectiveness over time and generations due to gene silencing. In terms of manipulating RNA silencing in specific cell types, the mobile nature of small RNAs can be an issue, which cautions against its use in the study of signaling and developmental processes that involve the propagation of local cell-to-cell or long-distance systemic signals.

Recent years have witnessed remarkable advances in developing methods for targeted genome engineering. Zinc finger nucleases (ZFN) and transcription activator-like effector nucleases (TALEN) have been employed in plants for targeted gene editing to improve important traits such as disease-resistance (Gaj et al. 2013). However, their application is limited by off-target effects and the complexity of the design of constructs. ZFN and TALEN have largely been replaced by the CRISPR/Cas9 (Clustered Regularly Interspaced Short Palindromic Repeats/CRISPR associated protein 9) system, which has revolutionized functional genomic studies and opened new horizons for molecular breeding (Chen et al. 2019).

The CRISPR/Cas9 system was originally derived from an adaptive immune system in bacteria that defends against phages and plasmids (Ishino et al. 1987; Jinek et al. 2012). It captures small DNA fragments from invading DNA molecules and incorporates them into the bacterial genome as CRISPR sequences. These sequences are transcribed into pre-CRISPR RNAs (crRNAs), which are processed into mature crRNAs with the help of trans-activating crRNAs (tracrRNAs). The crRNA and tracrRNA form a complex with the Cas9, generating an active endonuclease. The crRNA, adjacent to a protospacer adjacent motif (PAM) such as the NGG sequence, targets homologous viral DNA and induces double strand breaks (DSBs). This system was later adapted to be active in heterologous organisms for targeted genome editing, and further simplified by linking crRNA and tracrRNA into a single guide RNA (gRNA) (Doudna and Charpentier 2014; Hsu et al. 2014). Thanks to these improvements, the highly efficient CRISPR/Cas9 technology has rapidly become the most convenient and efficient gene editing tool, used across various organisms for editing either single or multiple targets (Cong et al. 2013; Doudna and Charpentier 2014; Jinek et al. 2012). It has ushered in a new era of functional genomic studies and applications in plants, including *Arabidopsis thaliana*, rice and wheat, as well as legumes (Bortesi and Fischer 2015; Jacobs et al. 2015).

Since 2015, numerous studies on gene editing in different legumes have highlighted significant advances in the field, using either composite plants generated via *Agrobacterium rhizogenes*-mediated root transformation or stable plant transformation with *A. tumefaciens* (Table 1). This review summarizes recent developments in legume genome editing, with a particular focus on cell-type-specific CRISPR/Cas9 techniques used to study the root nodule symbiosis.

**Table 1:** Non-exhaustive list of CRISPR/Cas9-mediated genome editing in legumes

Species and ecotype	Targeted locus	Transformant type	Editing efficiency	Cas9 system	gRNA promoter	Vector backbone	References
Medicago GUS transgenic line	*GUS*	Hairy root	NA	2Xpro35S–GmCas9	proAtU6-26	pMDC32	Michno et al. 2015
Medicago R108	*MtPHO2-Like*	Stable transgenic	50–70%	proGmUbi–Cas9	proAt7SL, proAtU6	pSC218GG/ pSC218GH	Curtin et al. 2017
Medicago R108	*MtNPD1/2/3/4/5*	Stable transgenic	NA	proGmUbi–Cas9	proCmYLCV	pSC218GG	Trujillo et al. 2019
Medicago Jemalong A17	*MtGA2ox10*	Hairy root	19%	pro35S–Cas9	proMtU6–8	pGK3304	Kim et al. 2019
Medicago R108	*MtPDS*	Stable transgenic	16.5–70%	pro35S, proEC1-2, proAtAMGE3, proAtUbi10–Cas9	proAtU6	pCAMBIA	Wolabu et al. 2020
Medicago Jemalong M9-10a	*MtCYP93E2*	Stable transgenic	84%	pAtUbi–Cas9	proAtU6-26	pDGB3 omega1	Confalonieri et al. 2021
Medicago R108	*MtCEP1/2/5/8/12*	Stable transgenic	48–60%	2Xpro35S–zCas9	proMtU6-1/3/5/6	pHSE401	Zhu et al. 2021
Medicago R108 (*Mtnlp2-1*)	*MtNIN*	Hairy roots	NA	proMtNCR158–zCas9	proAtU6-26	pKSE401	Jiang et al. 2021
Medicago Jemalong A17	*MtDRM2/L2*	Hairy root	NA	proGmUbi–Cas9	proMtU6-1, proMtU6-6,	Golden Gate binary vector	Pecrix et al. 2022
Medicago Jemalong A17	*MtAUR1*	Hairy root	NA	proMtChOMT3–zCas9	proAtU6-26	pKSE401	Gao et al. 2022
Medicago R108	*MtCLE35*	Stable transgenic	90%	pro35S–zCas9	proAtU6-26	pHSE401	Lebedeva et al. 2023
Medicago R108	*MtLYK10*, *MtMFS1/2*	Stable transgenic	70%	proGmUbi/pro35S-Cas9	proMtU6-1, proMtU6-26	pISV2678	Zhang et al. 2024a
Lotus MG20	*LjSYMRK*, *LjLb1/2/3*	Hairy root, Stable transgenic	35%	pro35S/proLjLb2–Cas9	proLjU6-1	pCAMBIA1300	Wang et al. 2016; Wang et al. 2019
Lotus MG20	*LjCZF1/2*	Hairy root	25–46%	2Xpro35S–hCas9	proLjU6-1	pCAMBIA1300	Cai et al. 2018
Lotus MG20	*LjBAK1*	Stable transgenic	NA	2Xpro35S–hCas9	proLjU6-1	pCAMBIA1300	Feng et al. 2021b
Lotus MG20	*proLjNIN-GA-RE*	Stable transgenic	NA	proZmUbi–FFCas9	proAtU6	pZH_gYSA	Akamatsu et al. 2021
Lotus MG20	*proLjNIN-CYC-RE*	Stable transgenic	NA	proZmUbi–FFCas9	proAtU6	pZH_gYSA	Akamatsu et al. 2022
Lotus MG20 (*Ljtml-4*)	*LjLHK1*	Stable transgenic	NA	proZmUbi–FFCas9	proAtU6	pZK_gYSA	Soyano et al. 2024
Soybean Jack-GFP	*GFP*	Hairy root	> 70%	pro35S–hCas9	proMtU6-6	p201N	Jacobs et al. 2015
Soybean Williams 82	*GmSHR*, *GmFEI*	Hairy root	0.6–95%	proZmUbi–dpCas9	proAtU6	NA	Cai et al. 2015
Soybean Williams 82	*Glyma06g14180, Glyma08g02290, Glyma12g37050*	Hairy root	3.2–20.2%	pro35S–Cas9	proAtU6-26, proGmU6-10	pCAMBIA3301	Sun et al. 2015
Soybean Williams 82	*GmmiR169c*,* GmNF-YA-C*	Hairy root, Stable transgenic	NA	proGmSCREAM–Cas9	proGmU6	pGES201	Xu et al. 2021
Soybean Williams 82	*GmROP6/9*	Hairy root	21–35%	pro35S–zCas9	proAtU6-26	pKSE401	Gao et al. 2021
Soybean Williams 82	*GmSTF3/4*	Stable transgenic	NA	NA	proGmU6	pCas9, pCAMBIA3301	Wang et al. 2021
Soybean Tianlong 1	*GmSTF1/2*	Stable transgenic	NA	proRPS5A–Cas9	proU6	JRH0645	Ji et al. 2022
Soybean Williams 82	*GmNIN1a/1b/2a/2b*	Stable transgenic	NA	proGmSCREAM–Cas9	proGmU6	pGES201	Fu et al. 2022
Soybean Dongnong 50	*GmmiR172c*	Hairy root	NA	pro35S–zCas9	proAtU6-26	pKSE401	Yun et al. 2023
Soybean Huachun 6	*GmUVR8*	Stable transgenic	NA	proGmSCREAM–Cas9	proGmU6	pGES201	Chen et al. 2024a
Soybean Williams 82	*proGmNF-YC4-1*	Stable transgenic	NA	proZmUbi–Cas9	proU6	pCas9–GW	Wang et al. 2024

*GmCas9* *Glycine max* codon‐optimized Cas9, *zCas9* *Zea mays* codon-optimized Cas9, *hCas9* human codon optimized Cas9, *FFCas9* fast and flexible Cas9, *dpCas9* dicotyledons codon-optimized Cas9, *NA* no annotation

## Genome editing in model legumes

### The applications of CRISPR/Cas9 in *Medicago truncatula*

The application of the CRISPR/Cas9 technology in *Medicago truncatula* (hereafter referred to as medicago) has significantly advanced our understanding of symbiotic nitrogen fixation. A first application of the CRISPR/Cas9 system in medicago was reported in 2015, where the β-glucuronidase (GUS) transgene was targeted in GUS transgenic plants through hairy root transformation (Michno et al. 2015). Later, CRISPR/Cas9 was employed to validate genome-wide association candidates controlling quantitative variation in nodulation using transgenic lines obtained by stable transformation (Curtin et al. 2017). Mutations through CRISPR/Cas9 of the gibberellic acid (GA)-inactivating C20-GA2-oxidase (*MtGA2ox10*) in transgenic hairy roots demonstrated its role in both early rhizobial infection and later stage nodule development (Kim et al. 2019). Using this technology, mutations were made in Domains Rearranged Methyltransferase (*MtDRM2* and *MtDRM2L2*), which are major players in DNA methylation impacting nitrogen-fixation activity and late stage nodule differentiation (Pecrix et al. 2022). Similarly, the *MtCLE35* gene, encoding a CLAVATA3/ESR-related peptide, was knocked out to investigate its local and systemic roles in regulating nodulation (Lebedeva et al. 2023).

One substantial advantage of CRISPR is the expedited generation of multiple mutants, a process that previously required labor-intensive identification, genotyping, propagation, and crossing of *Tnt1* transposon or EMS mutants. A family of five Nodule-specific Polycystin-1, Lipoxygenase, Alpha Toxin (PLAT) Domain genes (*MtNPD1/2/3/4/5*) was identified and confirmed to play a role in nodule organogenesis through multiple gene editing (Trujillo et al. 2019). The quintuple *Mtnpd1/2/3/4/5* knockout lines developed numerous but small ineffective white nodules.

Similarly, multigene editing of C-terminally Encoded Peptides *MtCEP1/2/5/8/12* has been achieved (Zhu et al. 2021)*.* These peptides are essential signaling molecules for regulating nodulation systemically from shoots (Laffont and Frugier 2024; Zhang et al. 2024b). This editing allowed generating a series of single, double, triple, and quintuple mutants, highlighting that *MtCEP1/2/12* act redundantly as the major long-distance signaling factors controlling nodule number (Zhu et al. 2021). These findings underscore the potential of CRISPR/Cas9 in unraveling complex or deeply redundant gene interactions, and notably for gene families clustered in the same chromosomal region for which the crossing of insertional mutants was impossible, such as in the case of *CEP* genes.

CRISPR/Cas9 was also used to target mutations in promoters. An interesting example of precise editing involves targeting the ‘*double Nitrate Responsive Element*’ (*dNRE*) motif in the promoter of the Leghemoglobin (*MtLb1*) gene in medicago (Jiang et al. 2021). This resulted in a strongly decreased expression of *MtLb1*, but not *MtLb2*, whose *dNRE* promoter region contained the gRNA target sequence but lacked the NGG–PAM sequence. This highlights the relevance of CRISPR/Cas9 for generating highly specific gene editing.

### Key factors affecting CRISPR/Cas9 editing efficiency in legumes

When comparing different CRISPR/Cas9-based studies deployed in legumes (Table 1), particularly in medicago, varying editing efficiencies among the different vectors used were observed. Interestingly, four different promoter-driven Cas9 systems targeting *Phytoene Desaturase* (*MtPDS*) genes were designed to compare their editing efficiency in medicago (Wolabu et al. 2020). This revealed a fourfold increase in mutation efficiency when the *Arabidopsis Ubiquitin* promoter (*proAtUbi10*) was used to drive *Cas9* expression, compared to the commonly used *Cauliflower Mosaic Virus* (CaMV) 35S promoter. Similarly, when targeting the Major Facilitator Superfamily transporter *MtMFS2*, a higher editing efficiency (70%) was achieved when using the soybean *Ubiquitin* promoter (*proGmUbi*) compared to a 50% efficiency using the same vector expressing *Cas9* from the 35S promoter (Zhang et al. 2024a). However, in another study, a CRISPR system designed with a *pro35S–Cas9* expression cassette achieved 84% editing efficiency when mutating the Cytochrome P450 monooxygenase *MtCYP93E2* (Confalonieri et al. 2021). Although different promoters have shown varying efficiencies in driving *Cas9* expression, it appears that any strong constitutive promoter is sufficient for routine applications.

The efficiency of editing is also influenced by other factors, including gRNA selection and the targeted genes. A recent review study found significant variability in the published gRNAs, including in legumes where gRNAs ranging from 15 to 22 bp in length (20 bp being the most common) and having a GC content of 35–70% (average 45%) were used (Jain et al. 2023). The presence of a G at the 5’ end of a gRNA (GN_19_-NGG) was found to be crucial for efficient genome editing (Gungor et al. 2023). A more efficient gene editing can be also achieved when two or more gRNAs are designed to target the same gene (Li et al. 2024). To evaluate the editing efficiency of different CRISPR elements, *Nicotiana benthamiana* leaves can serve as a test system to rapidly verify the functionality and gene editing activity of various CRISPR/Cas9 expression cassettes through transient transformation (Zhang et al. 2024a). Although results in *N. benthamiana* may not fully extrapolate to medicago, they can serve as an initial screening tool, with further optimization potentially needed to extrapolate to legumes.

The efficiency of CRISPR/Cas9 mediated mutagenesis can be alternatively also rapidly tested in transgenic roots of composite plants generated using the *A. rhizogenes*-mediated root transformation system, which takes 3–4 months. The combination of hairy root transformation and CRISPR/Cas9 has been thoroughly discussed and termed ‘hairy CRISPR’ (Kiryushkin et al. 2021), providing a rapid, precise, and efficient platform for functional gene analysis in legume plants. Despite these advantages, hairy roots are limited to root tissues, necessitating further efforts to produce stable transgenic lines and mutant seeds.

Noteworthy, stable transgenic plants can be regenerated from hairy roots in approximately 4.5–6.5 months from vector design to the recovery of plantlets with mutations (Zhang et al. 2020). However, biallelic mutations were found in only one of the 20 lines generated from hairy roots (Zhang et al. 2020), presenting a significant challenge for screening homozygous mutants compared to the classical regeneration procedure from *A. tumefaciens*-transformed leaf explants. Indeed, stable transformation from leaf explants takes at least 9 months to generate T0 plants. An improved protocol involving the infection of shoot bisection explants by *A. tumefaciens* was, however, set up for a faster regeneration of stable transgenic medicago (ecotype R108) plants, reducing the time to 5–6 months (Wen et al. 2019). Further optimization of stable transformation approaches to achieve high efficiency in the CRISPR/Cas9 system is still crucial for advancing legume research even faster.

### The applications of CRISPR/Cas9 in *Lotus japonicus*

*Lotus japonicus* (hereafter referred to as lotus) is an important model legume for studying beneficial microbial associations. In 2016, researchers successfully mutated the *LjSYMRK* (Symbiosis Receptor-Like Kinase) gene, a key player in symbiosis, using the CRISPR/Cas9 system for the first time in lotus, achieving a mutation efficiency of approximately 35% (Wang et al. 2016). Researchers later targeted *Leghemoglobin* (*Lb*) genes, which are essential for effective nitrogen fixation in root nodules (Larrainzar et al. 2020; Ott et al. 2005). The construction of *Ljlb1/2/3* triple mutants revealed the functional redundancy of these genes in maintaining the low oxygen environment in legume nodules (Wang et al. 2016, 2019 ). Similarly, mutations were introduced using CRISPR/Cas9 in the C3HC4-type RING zinc-finger *LjCZF1/2*, which interact with the cytokinin receptor Lotus Histidine Kinase LjLHK1 (Cai et al. 2018). Analysis of *Ljczf1/2* double knock-out roots revealed their function as positive regulators of nodulation. Later, the *LjBAK1* gene, encoding a brassinosteroid-related receptor kinase, was knocked-out to investigate the regulation of immunity during rhizobial infection, revealing that two *Ljbak1* mutants exhibited increased infection thread formation (Feng et al. 2021b). A recent study demonstrated the effect of periodic cytokinin responses on the rhizobium infection zone along the roots, through combined genetic analyses where *LjLHK1* was knocked-out by CRISPR/Cas9 in the *too much love Ljtml-4* mutant background (Soyano et al. 2024).

Nodule Inception (NIN) is a central transcription factor involved in symbiotic infection and nodule organogenesis (Shen and Feng 2024). The promoter of *NIN* contains numerous response elements that regulate its differential expression during nodulation (Hirsch et al. 2009; Liu et al. 2019; Xiao et al. 2020; Yoro et al. 2014). CRISPR/Cas9 has been instrumental in precisely targeting *cis*-elements within the promoter region of *NIN* to study the fine-tuning of its regulation. CYCLOPS directly regulates *NIN* expression via a *CYCLOPS Response Element* (*CYC-RE*) (Singh et al. 2014). The CRISPR/Cas9-mediated deletion of the *CYC-RE* in the promoter of *LjNIN* led to a significant reduction, but not a complete abolition, of infection thread formation, indicating the importance of the *CYC-RE* in fine-tuning *NIN* expression during rhizobial infection (Akamatsu et al. 2022). During nodulation, NIN directly activates a subset of GA biosynthetic genes (Gao et al. 2023), and conversely, GA induces the expression of *NIN* through a GA-responsive *cis*-acting region (*GA-RE*) in the *LjNIN* promoter (Akamatsu et al. 2021). Mutants with deletion of the *GA-RE* exhibit increased rhizobial infection and nodule number, along with a reduced GA-induced *CLE-RS1/2* (*CLE-Root Signal*) expression, suggesting that the inhibitory effect of GA is related to this systemic pathway.

Overall, in a short time period starting from 2015, the CRISPR/Cas9 technology has been efficiently used in both medicago and lotus through stable and transient root transformations to study symbiotic gene function. This approach provides a valuable complement to existing insertional mutant libraries and RNAi approaches, and has enhanced our ability to investigate and more precisely manipulate molecular symbiotic processes in legumes.

## Genome editing in legume crops

The application of CRISPR gene editing techniques in leguminous crops represents a significant advancement in both fundamental biological research and for enhancing sustainable agricultural practices. Soybean (*Glycine max*) is the most important legume crop, and traditional breeding through hybridization has always been a lengthy and laborious process.

Since 2015, several independent research groups have successfully edited genes in soybean hairy roots through CRISPR/Cas9, such as the *Short Root GmSHR* gene (Cai et al. 2015; Jacobs et al. 2015; Sun et al. 2015). However, the frequencies of indels (small insertions and deletions) varied widely, from 0.6% to 95%, largely due to differences in gRNA design. With improved gRNA design, the binary vectors delivering gRNA were further optimized by incorporating a native *GmU6* promoter to drive the *Cas9*, which increased mutation efficiencies (Sun et al. 2015). Subsequent studies utilized various Cas9 variants, including xCas9, Cas9–NG, and XNG–Cas9, to induce targeted gene mutations in hairy roots (He et al. 2022). These enzymes are able to recognize a range of NNG–PAMs, significantly expanding the scope of Cas9-mediated genome editing in soybean. Leveraging these optimized systems, an increasing number of studies have employed CRISPR to investigate the soybean–*rhizobium* symbiosis (Gao et al. 2021; Ji et al. 2022; Niazian et al. 2022; Wang et al. 2021; Xu et al. 2021; Yun et al. 2023).

Soybean is a diploid species that evolved from a paleotetraploid ancestor with a highly duplicated genome. This presents a challenge for conventional genetic approaches due to an increased functional redundancy. For example, the presence of four putative orthologous *GmNIN* genes in soybean complicates the detailed understanding of their individual roles. To effectively address such gene redundancy issues, recent advancements have introduced a pooled CRISPR/Cas9 platform designed for multiplex mutagenesis (Bai et al. 2020). By optimizing critical steps, such as utilizing a robust endogenous promoter *proGmSCREAM M4* to drive *Cas9* expression, effective gRNA assessment, and pooled transformation techniques, researchers have achieved an average mutagenesis frequency of 59.2% (Bai et al. 2020). Through this platform, a series of single, double, triple, and quadruple *Gmnin* mutants were generated, providing insights into the asymmetrically redundant function of *GmNIN* genes in soybean nodulation (Fu et al. 2022; Tu et al. 2024). Similarly, by targeting the ultraviolet B (UV-B) photoreceptor UV Resistance Locus 8 (*GmUVR8*), researchers recently generated *Gmuvr8a/b/c/d* single mutants, and a *Gmuvr8ac* double mutant to highlight the role of light signaling in nodulation and overall plant growth (Chen et al. 2024a).

The CRISPR technology has also been modified in order to induce single base pair substitutions (Anzalone et al. 2020). Two types of base editors, namely adenine base editors (ABEs) and cytosine base editors (CBEs), can mediate the substitution of adenine to guanine (A–G), and of cytosine to thymine (C–T), respectively. The CBE base editing tool has recently been deployed in soybean (Cai et al. 2020; Huang et al. 2023). In one study, researchers used a modified Cas9 nickase (Cas9n), a rat cytosine deaminase, and a uracil glycosylase inhibitor, to target *Flowering Locus T* (*FT*) homologs (Cai et al. 2020). Editing frequencies for *GmFT2a* and *GmFT4* were 18.2% and 6.0%, respectively. Notably, *GmFT2a*-edited plants exhibited both C-to-T and C-to-G mutations, while *GmFT4*-edited plants showed only C-to-G changes, suggesting that mutation types may depend on the sequence context or plant species (Cai et al. 2020). Recently, a new single-stranded DNA deaminase, named mini-Sdd7, achieved C-to-T editing efficiencies up to 67.4% in soybean hairy roots (Huang et al. 2023).

Base editing is ideal for modifying residues that are important for post-translational regulation, such as phosphorylation or proteolytic sites. This approach, for example, could be used to study NIN. Indeed, the NIN protein undergoes a proteolytic processing, generating a C-terminal fragment that regulates the maturation of the nodule to the nitrogen-fixing state (Feng et al. 2021a). Thus, a precise editing of NIN around the cleavage sites could provide insights into our understanding of NIN’s roles during nodulation. In addition, numerous agriculturally significant traits in soybeans are associated with single nucleotide polymorphism (SNP) variations (Liu et al. 2020). Using base editing tools to generate point mutations at functional SNPs is, therefore, likely to become a key strategy in molecular breeding.

Some of these genome editing efforts have also focused on promoting sustainable agricultural practices. For example, mutation of *Rhizobially Induced CLE* (*GmRIC1a/2a*) peptides in soybean increased nodule numbers and enhanced carbon and nitrogen acquisition, leading to higher grain yields and improved protein contents (Zhong et al. 2024). In addition, a recent study targeting the repressor *cis*-elements in the promoter region of *Nuclear Factor Y subunit C4* (*GmNF-YC4-1*) allowed increasing the expression of *GmNF-YC4-1*, resulting in a lower leaf starch content combined with a higher leaf protein content in soybean (Wang et al. 2024). These advancements underscore the potential of genome editing in engineering high yields and improved quality legume crops as well as decrease the use of fertilizers.

## Cell-type-specific CRISPR/Cas9 in legumes

### Advantages of cell-type-specific CRISPR in legume genome editing

**Fig. 1:**
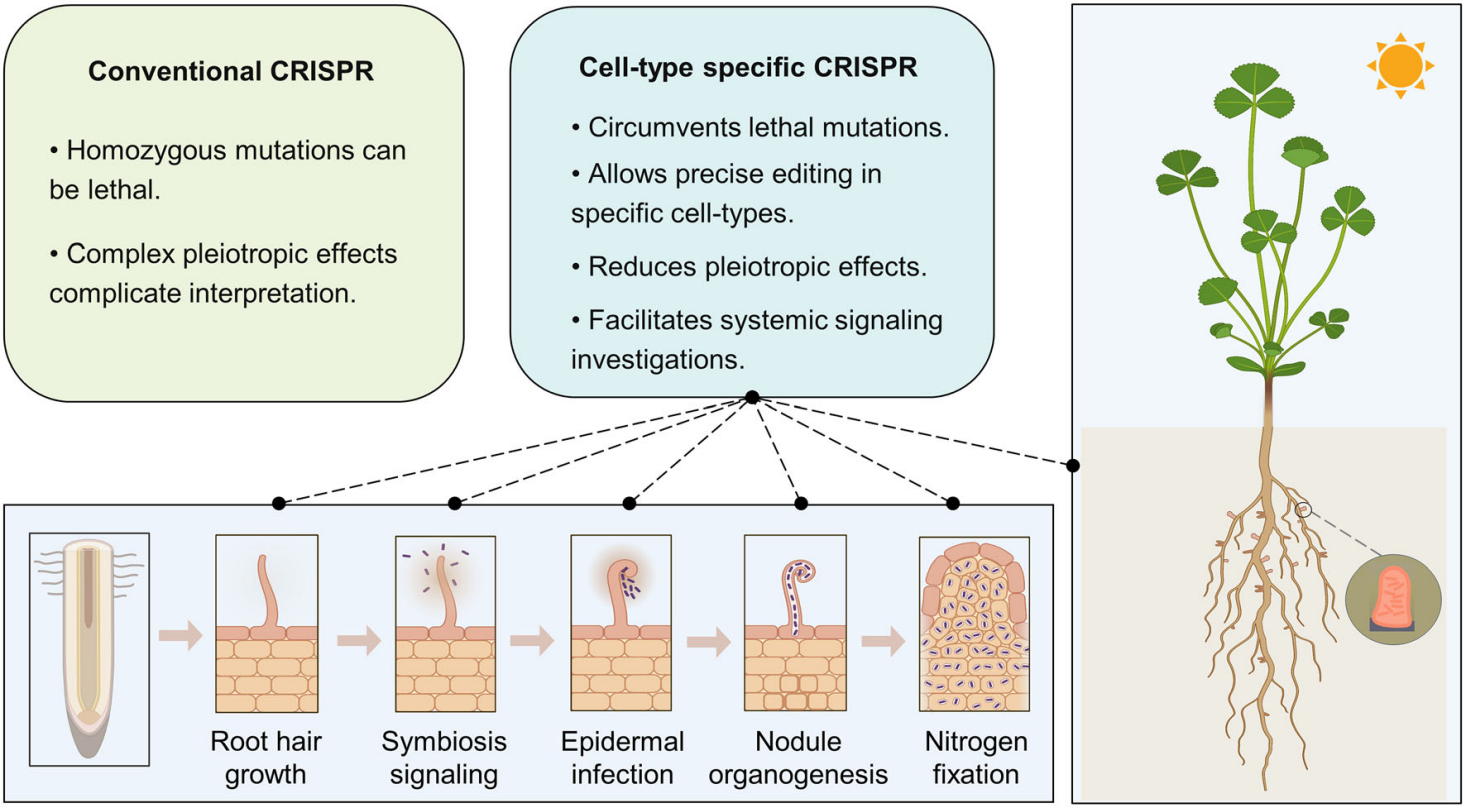
Advantages of cell-type-specific CRISPR in legume genome editing. The limitations of conventional CRISPR in the legume–rhizobia symbiosis are contrasted with the advantages of cell-type-specific CRISPR. This advanced technique will enable the precise manipulation of each symbiotic nodulation stage in different legume plants, ultimately helping to improve biological nitrogen fixation efficiency. Some of the images were created with BioRender (https://www.biorender.com)

More than 20 years of genetic study has yielded many genes with crucial and specific roles in the legume–rhizobia symbiosis (Jhu and Oldroyd 2023; Roy et al. 2020). Nonetheless, the function of hundreds of genes that are differentially expressed during nodulation remains unknown (Mergaert et al. 2020; Serrano et al. 2024). A frequent challenge in studying processes recruited for nodulation but that are also essential throughout the plant is that whole plant stable mutants might not be viable as homozygotes or may have pleiotropic effects that indirectly interfere with the interpretation of the symbiotic phenotype being studied (Fig. 1).

Auxin is essential for root growth and architecture, but also plays crucial roles in nodulation (Breakspear et al. 2014; Nadzieja et al. 2018; Ng et al. 2015). Using cell-type-specific CRISPR, auxin-related target genes could be knocked-out specifically during rhizobial infection or nodule organogenesis. This approach allows bypassing the additional effects of auxin on root hair and root growth. Another example is the investigation of the systemic signaling regulation of nodulation through the xylem and phloem vasculature (Gautrat et al. 2021; Laffont et al. 2020), for which cell-type-specific CRISPR knock-out targeted at these cell types would be a compelling approach to study further these whole plant systemic signals.

To overcome these limitations, recent advances in *Arabidopsis* genome editing have involved the construction of vectors in which tissue-specific or inducible promoters are used to drive *Cas9* expression (Decaestecker et al. 2019; Wang et al. 2020). This approach, termed CRISPR–TSKO (CRISPR-based Tissue-Specific Knock-Out system), enables the generation of mutations in particular plant cell types, tissues, and/or organs (Decaestecker et al. 2019). Recently, tissue/cell-specific CRISPR applications have also been explored in legume plants. These approaches, however, rely on identifying appropriate promoters, a task facilitated by the recent accumulation of cell-type-specific transcript profiling studies (Carrere et al. 2021; Mergaert et al. 2020) and single cell RNA-seq data gained at different stages of the symbiosis in different legumes (Cervantes-Perez et al. 2022; Frank et al. 2023; Liu et al. 2023a, b; Ye et al. 2024). Publicly available gene expression studies in *A. thaliana* are also valuable, as many *Arabidopsis* promoters can be directly deployed in legumes (Gavrilovic et al. 2016).

### Cell-specific knockout in the nitrogen-fixing zone of differentiated nodules

A first example of using cell-type-specific promoters to drive the *Cas9* expression is the *LjLb2* promoter in lotus nodules (Wang et al. 2016). This strategy was designed to achieve elevated *Cas9* expression levels specifically in the central metabolically active region of nodules, thus facilitating a more effective and tissue-specific gene knockout in transgenic roots.

Another pioneering study used the promoter of the *Nodule Cysteine-Rich* (*NCR*) *MtNCR158* gene to drive the expression of the *Cas9* in the distal part of medicago nodules, enabling a tissue-specific knock-out of *MtNIN* (Jiang et al. 2021). Researchers confirmed that *MtNIN* was knocked out in nodule cells but not in root cells. This resulted in decreasing a leghemoglobin gene expression, while wild-type number of nodules was still formed in successfully edited plants. These findings demonstrate the synergistic regulatory roles of NIN-Like Protein 2 (NLP2) and NIN in leghemoglobin gene expression during symbiotic nitrogen fixation.

Like NIN, other important symbiotic regulators including *Nodulation Signaling Pathway 1* (*NSP1*) and *NSP2*, are required for rhizobial infection (Hirsch et al. 2009; Kalo et al. 2005; Smit et al. 2005) but are also expressed in mature nodules, making it difficult to study their roles only at these latter symbiotic stages. The use of a *Mtnsp2-3* weak allele allowed, however, revealing a late nodulation phenotype, with fewer uninfected cells, starch granule accumulation, and a delayed nodule differentiation (Kovacs et al. 2021). These data strongly suggest that the use of a cell-type-specific CRISPR would be an alternative approach for uncovering new roles of many “early nodulin” genes, such as *NSP1* and *NSP2*, specifically during the late stages of nodulation.

### Cell-specific knockout at the early stage of the legume–rhizobia symbiosis

The Nod factor receptor Nod Factor Perception (NFP) is essential for activating the symbiotic signaling in medicago (Oldroyd 2013), with corresponding mutants being unable to form any infection thread and nodule (Amor et al. 2003; Arrighi et al. 2006). A recent study used the *Chalcone O-methyltransferase MtChOMT3* promoter, which preferentially expresses in root cells infected by rhizobia (Chen et al. 2015), to drive the *Cas9* and achieve a targeted knock-out of *NFP* in medicago transgenic roots (Gao et al. 2022). Researchers used laser-capture microdissection to confirm that *NFP* was knocked-out specifically in the rhizobia-infected cells but not in uninfected cells. Consequently, a decreased formation of infection threads and nodules was observed in *NFP–TSKO* transgenic roots. Although nodulation was not completely inhibited in these edited roots, despite reaching an approximately 90% editing efficiency using 2 gRNAs, it was, however, enough to block most symbiotic infections and strongly restrict nodule formation. This CRISPR–TSKO strategy thus provides a mean to study genes that have a high “basal” expression in roots, allowing separating their roles in root development from a specific role in symbiosis.

A similar approach also enabled the study of the Aurora kinase 1 (MtAUR1) that is induced in infected root hair cells (Breakspear et al. 2014). AURs are integral to plant development through their regulation of the cell cycle, ensuring that cell division and differentiation occur at the correct time and location to support plant growth (Weimer et al. 2016). A null mutant of *MtAUR1* is thus lethal, but knocking-out *MtAUR1* specifically in rhizobia-infected cells with a *proMtChOMT3–Cas9* construct revealed abnormal morphologies of infection threads, indicating its function in facilitating the endosymbiosis of rhizobia and promoting bacterial colonization within plant cells (Gao et al. 2022). Thus, such CRIPSR–TSKO strategies enabled a precise gene editing in rhizobia-infected cells, circumventing the lethal or pleiotropic phenotypes that can arise from a whole plant gene knock-out. Interestingly, besides MtAUR1, numerous other cell cycle components have been hypothesized to play crucial roles in the symbiotic infections with rhizobial bacteria and mycorrhizal fungi (Batzenschlager et al. 2023; Breakspear et al. 2014; Russo et al. 2019). This CRIPSR–TSKO approach would likely be optimal to study their function specifically during symbiotic interactions.

### Bottlenecks and future strategies of cell-type-specific CRISPR

The above studies demonstrated that cell-type-specific CRISPR can be efficiently used for gene knock-outs in transgenic roots, resulting in an efficient editing of target loci, and altered early or late nodulation phenotypes.

**Fig. 2:**
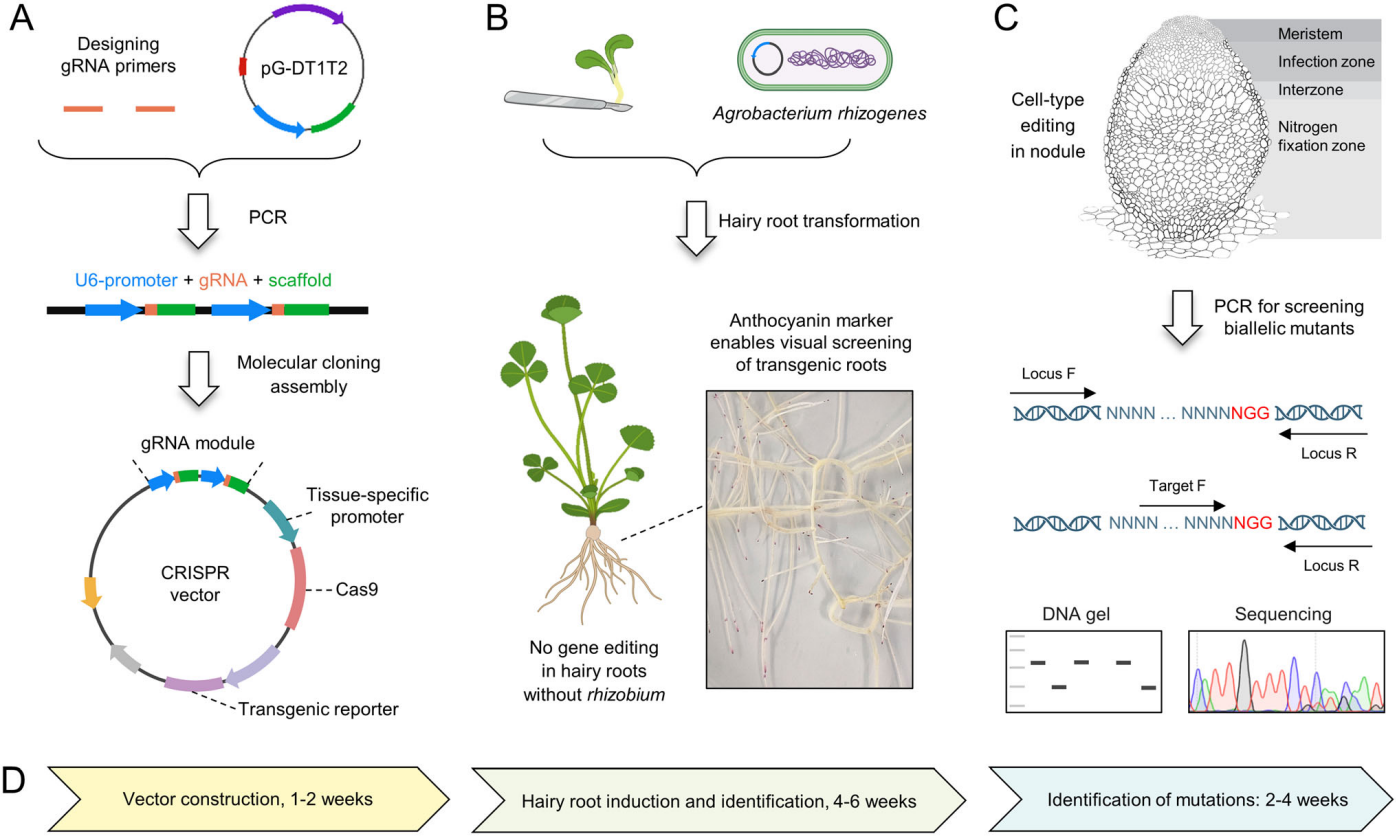
Workflow for generating cell-type-specific CRISPR in hairy roots. **A** Construction of vectors using standard molecular cloning assembly. **B** Transformation of *Medicago truncatula* hairy roots and identification of transgenic roots using anthocyanin as a visual marker. The red color indicates positive transgenic roots. It is expected that no gene editing occurs in plants in the absence of rhizobia due to the very low baseline of *Cas9* expression. **C** Identification of edited mutations using the Mutation Site-Based-Specific Primers PCR (MSBSP–PCR) strategy and Sanger sequencing after rhizobia inoculation. Genomic DNA from plant roots is purified and subjected to a PCR amplification using Locus-F/Target-F as forward primers, and Locus-R as reverse primer. The Locus-primers are designed to anneal 200–300 bp upstream and downstream from the PAM site, respectively, and the Target-F is a mutation site-specific primer that expands to the recognition site of the gRNA. Wild-type plants and heterozygous mutants will produce an amplification product, while homozygous/biallelic mutants will not show any amplification product due to edited sequences. **D** Timeline for obtaining biallelic mutant roots. The entire process takes approximately 2–3 months

One challenge in *A. rhizogenes*-mediated hairy root transformation is the coexistence of both transformed and untransformed roots, as well as of chimeric roots. Identification of positive transgenic hairy roots commonly employs markers such as GUS, GFP, and DsRed. Recently, anthocyanins and betacyanins have emerged as visible markers for monitoring gene expression and plant transformation efficiency (Chen et al. 2024b; He et al. 2020; Ruan et al. 2021). Expression of *MtLAP1* (*Legume Anthocyanin Production 1*) under a root-cap-specific promoter resulted in anthocyanin accumulation in the root tips of transgenic roots which can be easily evaluated by the naked eye (Ruan et al. 2021). This thus allows the easy and quick visual screening of transgenic roots without the use of histochemical staining or fluorescent microscopy (Fig. 2).

Achieving 100% editing efficiency using CRISPR is unlikely and it is even more unlikely that all edits will be homozygous. The Mutation Site-Based-Specific Primers PCR (MSBSP–PCR) approach offers a simple and cost-effective method for screening CRISPR/Cas9-induced homozygous/biallelic mutants (Guo et al. 2018). Using specific primers that target predicted DSBs and expand to the NGG–PAM, this technique allows to efficiently discriminate between wild-type plants (non-edited), which produce an amplification product, and homozygous/biallelic mutants, which do not exhibit any amplification due to the mutation (Fig. 2). Using this workflow, the entire process—from vector construction to obtaining biallelic mutation—can be completed in approximately 2–3 months. This streamlined design will help improving the throughput of using cell-type-specific CRISPR in legumes.

Several challenges remain, however, including off-target effects and variable gRNA efficiencies, both of which depend on the genes being targeted and the plant species (Grünewald et al. 2019; Peterson et al. 2016; Wienert et al. 2019). For applications dedicated to improving agricultural breeding, off-target effects may not be a concern as long as no unwanted phenotypic change is generated. The variable nature of the resulting mutation can also be a disadvantage, as not all induced mutations are loss-of-function (knock-out). It may be possible to avoid generating random mutations using a recent advanced methodology called CRISPR prime editing (Anzalone et al. 2019; Lin et al. 2020). This “search-and-replace” base editing technology allows researchers not only to choose the mutation desired, but also to generate specific amino-acid substitutions. Recent advancements in tomato and *Arabidopsis* have improved editing efficiency to 9.7% at the callus stage, and up to 38.2% in heritable alleles (Vu et al. 2024). However, the current inefficiency of prime editing in dicotyledonous plants remains a significant limitation (Sun et al. 2024), and this new technology has so far only been deployed in peanut, chickpea, and cowpea protoplasts (Biswas et al. 2022). Future adaptation of this powerful tool in dicotyledonous plants, including legumes, would present a significant opportunity for the symbiosis research field.

Another consideration is that the activation of the *Cas9* late in the development of an organ would be predicted to result in a mixture of genotypes. Depending on the editing efficiency, this may still allow producing useful genotypes. For example, researchers observed a range of infection phenotypes in the *Mtaur1* mutants generated by CRISPR–TSKO, where the *Cas9* expression was limited to root infected cells, suggesting that editing might have occurred at different times (Gao et al. 2022). Such a temporal editing window could likely be narrowed down using multiple gRNAs for a single target gene. Increasing the number of gRNAs was indeed shown to raise the editing efficiency in *Arabidopsis* (Li et al. 2024). Interestingly, a scalable vector has been recently developed in medicago, which can accommodate as least nine gRNAs (Lawrenson et al. 2023). Other strategies include the targeting of multiple genes, notably through the use of a polycistronic–tRNA–gRNA strategy expressed from a unique promoter to generate many gRNAs (Kim et al. 2021). Such multiplexed CRISPR technologies, where numerous gRNAs or Cas enzymes are expressed simultaneously, have facilitated powerful biological engineering applications in both animals and rice (McCarty et al. 2020). To assess the efficiency of different vector systems in legumes, *NFP* would provide a useful proof-of-concept, since it is straightforward to evaluate the non-nodulating phenotype (Nod-) induced in transgenic hairy roots.

Finally, the highly polyploid nitrogen-fixing cells of nodules (up to 64N) represent challenges for reaching an efficient editing efficiency because of the technical difficulty to identify mutations. For example, from a single *MtNIN–TSKO* nodule, researchers identified DNA sequences showing complex multi-peaks around the PAM site, indicating that multiple types of edits had occurred within the same nodule (Jiang et al. 2021). In such cases, the characterization of the target protein levels might be the most instructive way to determine the efficacy of the gene knock-out.

## Conclusion and perspectives of cell-type-specific CRISPR/Cas9

CRISPR-based genome editing techniques have emerged as powerful tools in legume biology and symbiosis research. Recent findings in transgenic roots suggest that the cell-type-specific CRISPR/Cas9 strategy is effective for investigating nodulation phenotypes in legume plants. As an alternative to the composite plant strategy already used, cell-type-specific CRISPR/Cas9 should be also used in stable transgenic lines. This will allow generating seeds carrying the CRISPR construct, for example *proMtNCR158–Cas9*, together with gRNAs targeting gene(s) of interest. The resulting stable plants would only express the *Cas9* upon nodulation, leading to the formation of edited nodules without affecting other plant organs. This approach is nonetheless only workable for promoters that are not expressed in cell lineages that give rise to the germline, and thus will be more likely to be effective for promoters that have a high tissue specificity.

Overall, the cell-type-specific CRISPR technology enables precise editing of genes and promoter regions in specific plant cell types, tissues, and organs. This approach reduces pleiotropic effects and can allow the fine-tuning of local and systemic symbiosis signals, optimizing nodulation and growth to ultimately enhance sustainable legume breeding. With sufficiently high editing efficiencies, this approach, in combination with prime editing, is anticipated to revolutionize plant genetics and to participate in achieving the ambitious goal of transferring symbiotic nitrogen fixation in non-leguminous plants in a foreseeable future.

## Data Availability

Data sharing not applicable to this article as no dataset was generated or analysed during the study.
